# Supplementary material for: Characterization of the MADS-Box Gene Family in Akebia trifoliata and Their Evolutionary Events in Angiosperms
Source: Genes (Basel). 2022 Oct 1;13(10):1777. doi: 10.3390/genes13101777 (PMC9601569; doi:10.3390/genes13101777)
Supplement: Supplementary file 1 [file genes-13-01777-s001.zip › Supplementary Figures.pdf]

**Supplementary Figures**

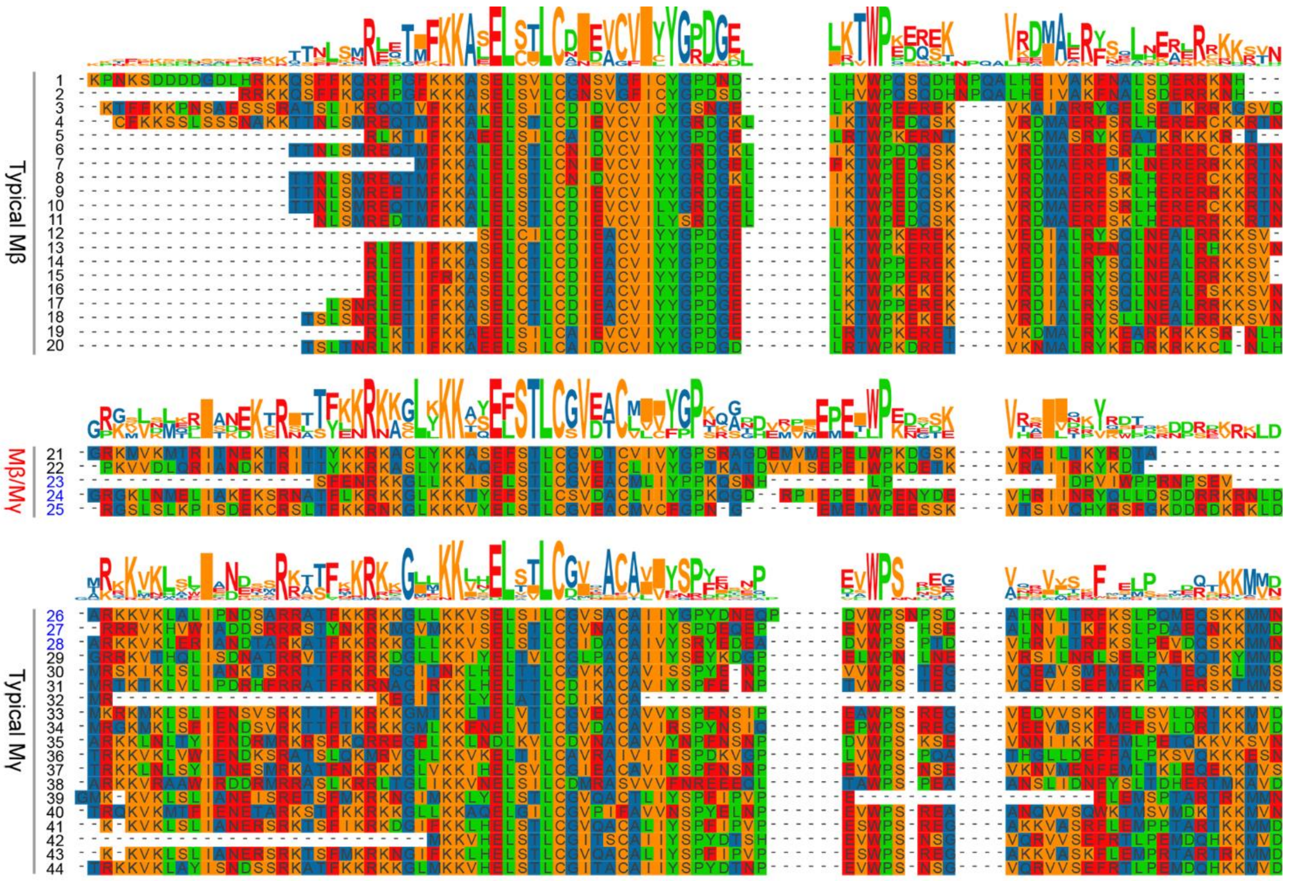


**Figure S1** : Comparison of MADS-box domains between Mβ and Mγ. The domains were annotated by NCBI CDD database and aligned by ClastalW. 1-20 represent typical *Arabidopsis* Mβ sequences, 21, 20 represent *AT5G55690* and *AT5G58890* sequences, 23-25 represent *EVM0009117*, *EVM0013722*, *EVM0016918* sequences. 26-44 represent typical *A. trifoliata* and *Arabidopsis* Mγ sequences.

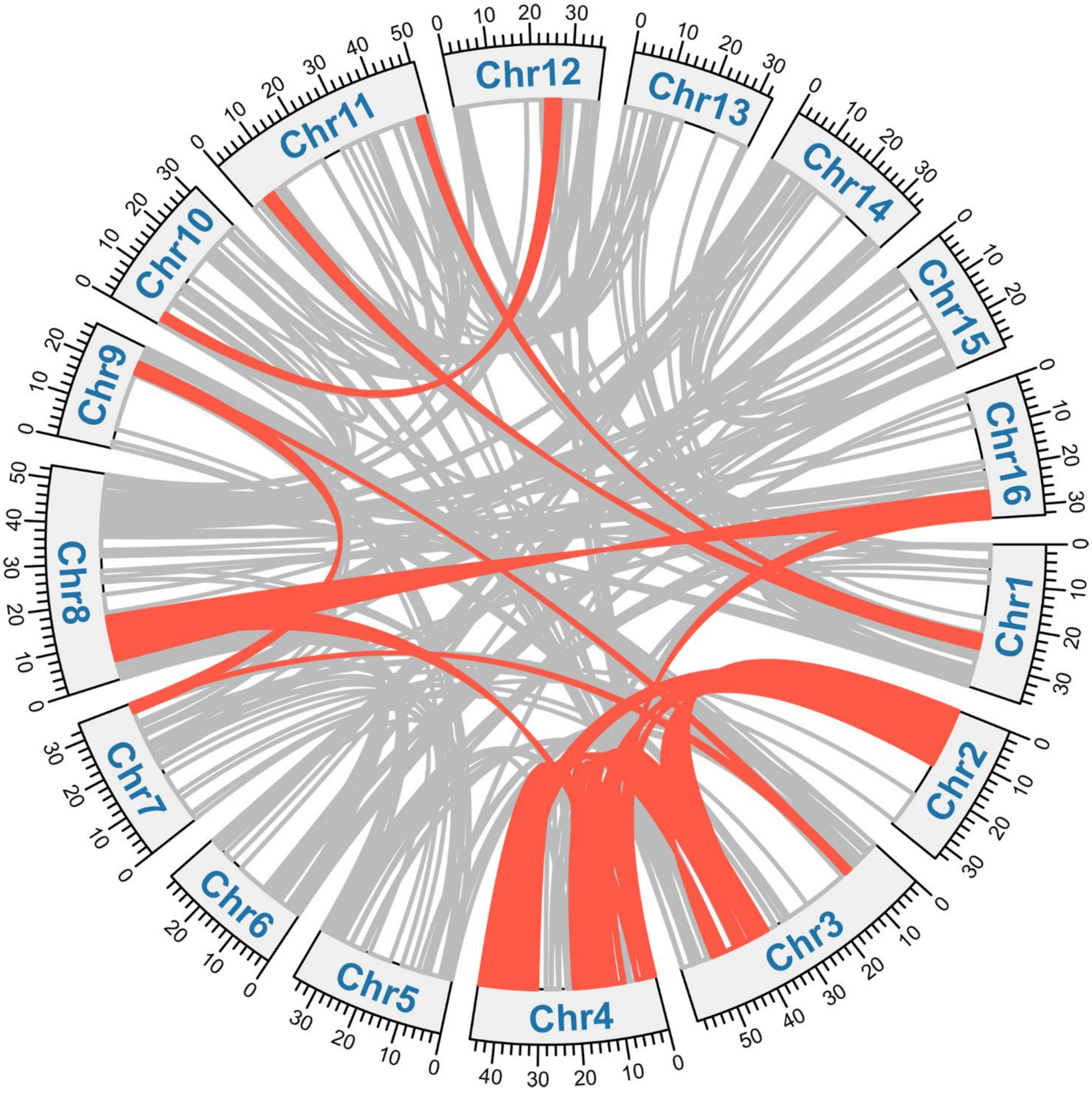


**Figure S2**. Synteny results of the *A. trifoliata* genome and MADS-box genes. Blocks in red contain MADS-box genes.

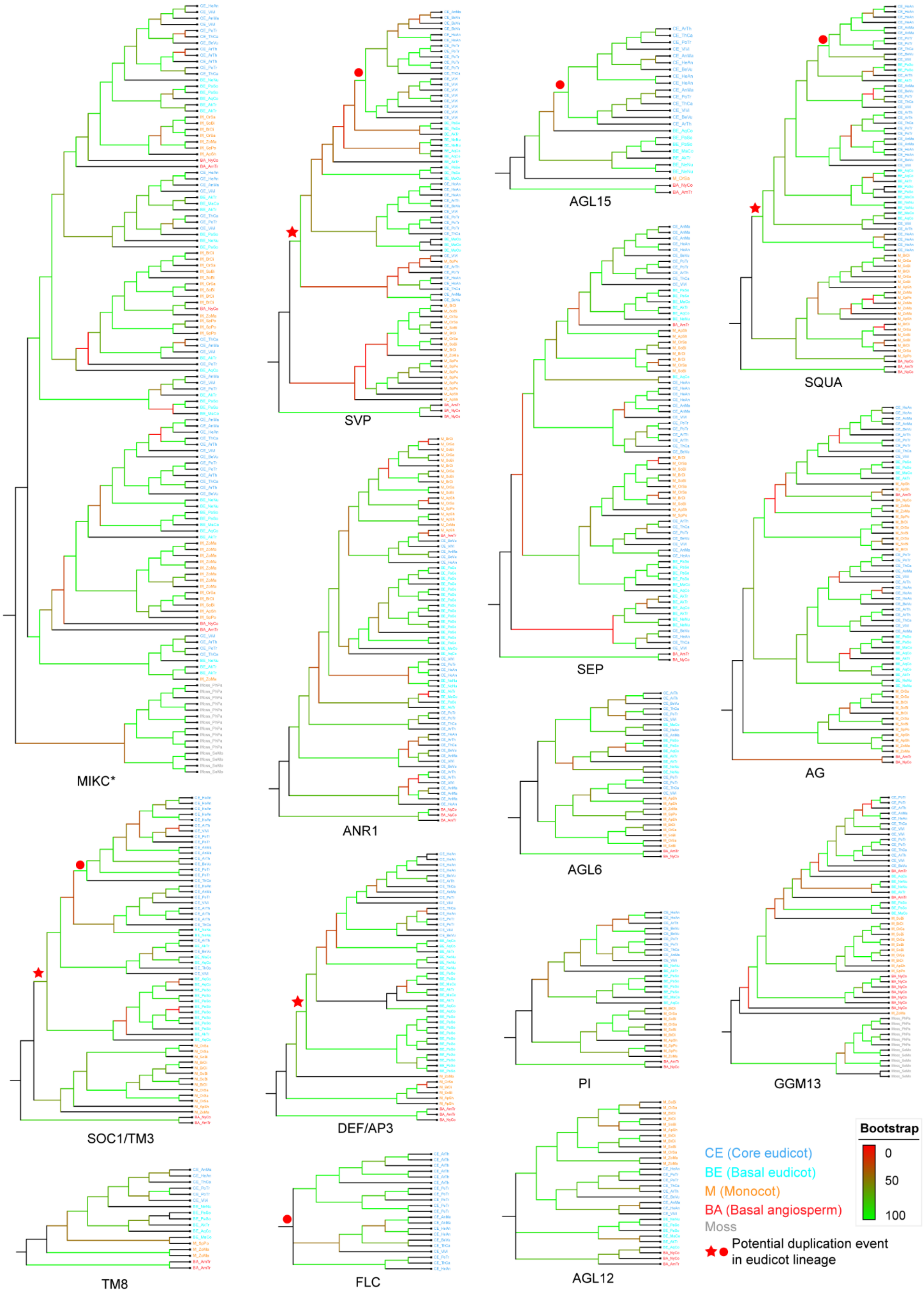
MIKC*
SVP
AGL15
SQUA
SEP
ANR1
AG
AGL6
SOC1/TM3
DEF/AP3
PI
GGM13
TM8
FLC
AGL12
CE (Core eudicot)
BE (Basal eudicot)
M (Monocot)
BA (Basal angiosperm)
Moss
Potential duplication event in eudicot lineage
Bootstrap
0
50
100

**Figure S3**. Phylogenetic tree of type II genes from 22 plant species. The color for each interior branch is the percent bootstrap value (1000 iteration). Detail of species and MADS-box genes are present in Table S4.
